# Wound Complication and Neuropraxia of the Posterior Cutaneous Nerve of the Arm after Primary Repair of a Latissimus Dorsi and Teres Major Tear

**DOI:** 10.1155/2022/7373178

**Published:** 2022-05-13

**Authors:** Matthew G. Alben, Neil Gambhir, Michael A. Boin, Kirk A. Campbell, Mandeep S. Virk

**Affiliations:** ^1^Division of Shoulder and Elbow Surgery, Department of Orthopedic Surgery, NYU Grossman School of Medicine, NYU Langone Orthopedic Hospital, NYU Langone Health, New York, NY, USA; ^2^Division of Sports Surgery, Department of Orthopedic Surgery, NYU Grossman School of Medicine, NYU Langone Orthopedic Hospital, NYU Langone Health, New York, NY, USA

## Abstract

We present a case of a surgically treated latissimus dorsi (LD) and teres major (TM) tear with a one-year outcome. The postoperative course was complicated by wound dehiscence requiring operative intervention and neuropraxia of the posterior cutaneous nerve of the arm. The report highlights previously unreported surgical risks associated with repair of LD/TM tendons.

## 1. Introduction

Tears of the latissimus dorsi (LD) and teres major (TM) are relatively uncommon and are typically observed in professional and high-level recreational athletes. Insertion of the LD and TM away from the humerus' axis of rotation subjects these tendon units to extreme forces in activities like overhead sports and cable watersports [1]. While LD and TM tears typically do not impair a patients' baseline activity, they may affect performance in throwing, climbing, or heavy lifting activities.

There is no consensus regarding ideal treatment of LD/TM tears as both surgical and nonsurgical interventions have been reported with comparable outcomes [2, 3]. As such, surgical repair offers improvements in shoulder girdle strength (in adduction and extension). Herein, we describe the postoperative complications and their treatment, in addition to the one-year outcome of a surgically treated patient with a tear of the LD and TM (Table 1). Consent was provided by the patient for use of data concerning their case to be submitted for publication.

## 2. Case Report

### 2.1. History and Preoperative Findings

**Figure 1:**
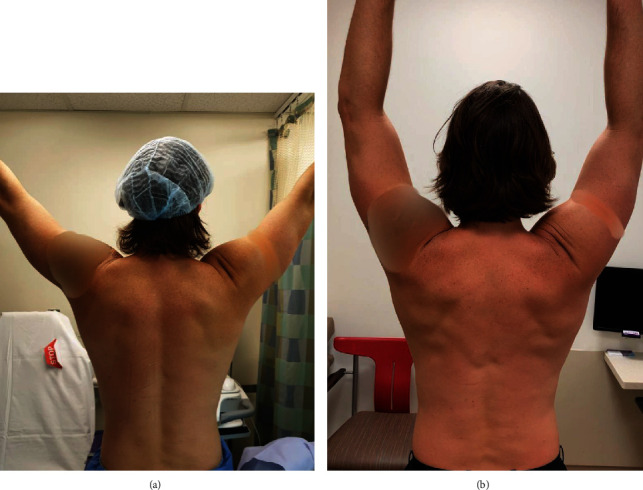
Clinical photographs: preoperative clinical photograph (a), six weeks after injury, demonstrating some residual left hemithoracic and axillary swelling with ecchymosis and a less conspicuous posterior axillary fold. Postoperative clinical photograph (b), one year after surgery, demonstrating symmetric contour of the left axillary fold and restoration of muscle bulk of the latissimus dorsi and teres major.

**Figure 2:**
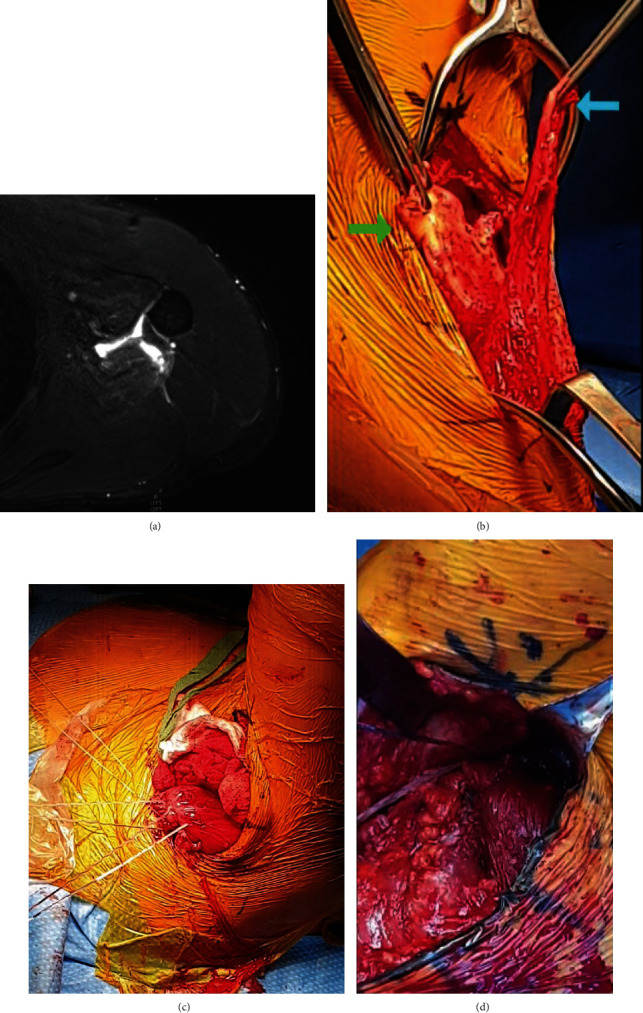
Edema and fluid are noted along the medial aspect of the proximal humerus, originating near the medial margin of the distal bicipital groove. There is a full-thickness, retracted tear of the latissimus dorsi humeral attachment, with retraction of tendon fibers approximately 2.5 cm medially and 2 cm distally. The teres major humeral attachment shows irregularity along its inferior margin (a). Intraoperative pictures demonstrating the torn latissimus dorsi (right arrow) and teres major (left arrow) tendons after adequate mobilization (b). The two tendon ends were secured together in a conjoint fashion using high-strength nonresorbable sutures in a Krackow fashion (c). The footprint was prepared, and sutures were passed through the cortical button and inserted into the medullary canal via unicortical drill holes. The tendon was reduced to the footprint using the sliding suture technique (d).

The index patient is a 31-year-old right-handed dominant male who presented after a wakeboarding accident in which he heard an audible pop accompanied by sharp left shoulder pain after a sudden pull by a tow cable. Physical examination was remarkable for extensive ecchymosis around the posterior aspect of the arm and left hemithorax, as well as swelling in the posterior axillary region (Figure 1(a)). Magnetic resonance imaging demonstrated a retracted full-thickness tear of the LD tendon and irregularity of the TM humeral footprint (Figure 2(a)). During the initial trial of nonsurgical treatment, the patient recovered full range of motion (ROM) in a pain-free arc but noted weakness during chin ups and weight lifting. Due to persistent weakness, he elected for surgical repair six weeks after injury.

### 2.2. Surgical Treatment and Postoperative Care

Primary repair of the LD and TM tendons was performed in a sloppy lateral position via a single axillary-based incision (Video 1). The posterior cutaneous branch (PCN) of the radial nerve was identified and protected during subcutaneous dissection. Deep dissection demonstrated a seroma covering the stumps of the torn LD and TM tendons, which were avulsed off their insertion from the bicipital groove.

Both tendons were mobilized and able to reach their insertional footprint for a direct primary repair (Figure 2(b)). Two high-strength, nonresorbable sutures were placed in a Krackow fashion to run up and down two inches of the tendon lengths (Figure 2(c)). The insertional footprint in the bicipital groove was decorticated, and two unicortical drill holes (3.5 mm, 2 cm apart) were made. The tendon was secured to the insertional footprint using two cortical buttons (Figure 2(d)). There was no gap at the repair site, and the long head of the biceps tendon was not incarcerated in the repair.

Postoperatively, the arm was placed in a sling for six weeks. Active adduction and internal rotation, passive overhead abduction, and external rotation were not permitted during this initial phase of immobilization. Active ROM was started at six weeks with the introduction of LD and TM strengthening exercises at three months.

### 2.3. Complications and Patient Outcome

The patient reported serous drainage from the incision and was found to have wound dehiscence in the central part of the incision (1 cm) three weeks after surgery. He underwent irrigation and debridement in the operating room with primary wound closure, and the incision healed uneventfully thereafter. The patient also reported decreased sensation in the distribution of the PCN of the arm. The sensation progressively improved throughout the year after surgery. The primary repair and PCN of the arm were both intact upon observation during the debridement surgery.

At one-year follow-up, the patient reported no pain and was able to return to preinjury sporting activity without limitations. He had normal contour of the left posterior axillary fold and full shoulder ROM (Figure 1(b)). He rated his subjective shoulder value to be 90% of his original function.

## 3. Discussion

Surgical repair of LD and TM offers improvement in strength in adduction and extension, as well as cosmetic improvement in the posterior axillary fold contour. Despite these advantages, the close proximity of the surgical site to neurovascular structures poses an inherent risk for injury [4]. In this report, we describe two previously unreported complications after surgical repair of the LD/TM. Though wound dehiscence can happen with any surgery, we believe that axillary-based incisions carry a high risk for such a complication as has been shown with other LD tendon surgeries. Stein et al. reported a wound dehiscence rate of 7.7% (6/78) after LD flap breast reconstruction using an analogous axillary incision [5]. In a cohort of 67 patients who underwent LD tendon transfer for the treatment of irreparable rotator cuff tears, Gerber et al. reported wound dehiscence in one patient [6].

The PCN of the arm is encountered in an axillary-based approach for LD repair and is at risk for injury. However, this complication has not been previously reported with LD/TM tendon repairs. As the presentation for PCN of the arm neuropraxia can vary and potentially be underreported, awareness of this complication is pertinent to patients undergoing repair and those treating them. Previously reported neuropathies in studies involving LD tendon transfers include those of the axillary nerve, ulnar nerve, and brachial plexus injury [6]. In our index case, the PCN was intact and the neuropraxia improved without any additional intervention.

## 4. Conclusion

There continues to be debate regarding ideal treatment of LD/TM tendon tears. Surgical treatment is an acceptable treatment option for combined LD and TM tendon tears, but patients must be counseled for potential risks and complications. Although there is paucity of literature on surgical outcomes, wound complications and neurological injuries are important risks in addition to retear or failure of repair.

## Figures and Tables

**Table 1:** Patient-reported outcomes.

	Surgical (12 months)
PROMIS upper extremity	46.6 ± 3.0
PROMIS pain interference	51.1 ± 1.9
PROMIS pain intensity	40.5 ± 2.8
PROMIS general life satisfaction	74.6 ± 4.4
American Shoulder and Elbow Surgeons score	80
Subjective shoulder value	90
*PROMIS*: Patient-Reported Outcome Measurement Information Systems	

The patient-reported outcome measures at the patient's 12-month follow-up visit.

## Data Availability

No archived datasets were relevant to the preparation of this manuscript.

## Supplementary Materials

Supplementary Materials Included in this case report are the preoperative and one-year postoperative clinical photographs, in addition to the preoperative MRI and intraoperative images as referenced in the surgical treatment. Additionally, we have included a video of our surgical technique. Video 1. 0:00-0:13: introduction and disclosures. 0:14-0:20: preoperative imaging. 0:21-0:54: axillary incision and subcutaneous dissection with identification of the posterior cutaneous nerve. 0:55-1:26: identification and isolation of the torn latissimus dorsi and teres major tendons from each other and surrounding fascia. 1:27-1:46: the two tendon ends were secured together in a conjoint fashion using high-strength nonresorbable sutures in a Krackow fashion, which were then threaded through two unicortical button devices. 1:47-2:36: the bicipital groove footprint for the two tendons was debrided and two unicortical drill holes were made. The tendons were reduced to the footprint using the sliding suture technique. 2:37-2:44: irrigation and closure. 2:45-2:56: postoperative management and rehabilitation. 2:57-3:19: postoperative outcome. 3:20-4:26: postoperative clinical pictures at 12-month follow-up.
