# Supplementary material for: Wound Complication and Neuropraxia of the Posterior Cutaneous Nerve of the Arm after Primary Repair of a Latissimus Dorsi and Teres Major Tear
Source: Case Rep Orthop. 2022 May 13;2022:7373178. doi: 10.1155/2022/7373178 (PMC9122720; doi:10.1155/2022/7373178)
Supplement: Supplementary Materials — Included in this case report are the preoperative and one-year postoperative clinical photographs, in addition to the preoperative MRI and intraoperative images as referenced in the surgical treatment. Additionally, we have included a video of our surgical technique. Video 1. 0:00-0:13: introduction and disclosures. 0:14-0:20: preoperative imaging. 0:21-0:54: axillary incision and subcutaneous dissection with identification of the posterior cutaneous nerve. 0:55-1:26: identification and isolation of the torn latissimus dorsi and teres major tendons from each other and surrounding fascia. 1:27-1:46: the two tendon ends were secured together in a conjoint fashion using high-strength nonresorbable sutures in a Krackow fashion, which were then threaded through two unicortical button devices. 1:47-2:36: the bicipital groove footprint for the two tendons was debrided and two unicortical drill holes were made. The tendons were reduced to the footprint using the sliding suture technique. 2:37-2:44: irrigation and closure. 2:45-2:56: postoperative management and rehabilitation. 2:57-3:19: postoperative outcome. 3:20-4:26: postoperative clinical pictures at 12-month follow-up. [file 7373178.f1.docx]

January 26th, 2022

Dear Editor,

We wish to submit the surgical video with our original case report titled “Wound Complication and Neuropraxia of the Posterior Cutaneous Nerve of the Arm After Primary Repair of a Latissimus Dorsi and Teres Major Tear”. As we were unable to upload the video file with the submission directly, the link below will direct to the associated video. Let us know if there’s a preferred method to upload this video.

<https://nyulangone-my.sharepoint.com/:f:/g/personal/matthew_alben_nyulangone_org/EsY5Nl8L8btLjTaMUo4zT78BtEenMvh1zzcCjLdMeGpHoA?e=essd16>

Please address all correspondence concerning this manuscript to me at [mandeep.virk@nyulangone.org](mailto:mandeep.virk@nyulangone.org).

Thank you for your consideration of this manuscript.

Sincerely,

Mandeep S. Virk, MD

Associate Professor, Department of Orthopedic Surgery at NYU Grossman School of Medicine
